# The mosaic accessory gene structures of the SXT/R391-like integrative and conjugative elements derived from *Vibrio* spp. isolated from aquatic products and environment in the Yangtze River estuary, China

**DOI:** 10.1186/1471-2180-13-214

**Published:** 2013-09-30

**Authors:** Yuze Song, Pan Yu, Bailin Li, Yingjie Pan, Xiaojun Zhang, Jian Cong, Yinying Zhao, Hua Wang, Lanming Chen

**Affiliations:** 1 Key Laboratory of Quality and Safety Risk Assessment for Aquatic Products on Storage and Preservation (Shanghai), China Ministry of Agriculture, Engineering Centre for Quality Control and Risk Assessment of Aquatic Products, College of Food Science and Technology, Shanghai Ocean University, 999 Hu Cheng Huan Road, Shanghai 201306, PR China; 2 Shanghai Center for Systems Biomedicine, School of Life Sciences and Biotechnology, Shanghai Jiao Tong University, 800 Dongchuan road, Minhang Campus, Shanghai 200240, PR China; 3 The Ohio State University, 2015 Fyffe Court, Columbus, OH 43210, USA

**Keywords:** *Vibrios*, ICEs, Drug and heavy metal resistance, Aquatic products, Estuary

## Abstract

**Background:**

The emergence, resurgence and spread of human food-borne pathogenic *Vibrios* are one of the major contributors to disease burden and mortality particularly in developing countries with disputable sanitary conditions. Previous research on pathogenic *Vibrio cholerae* and *Vibrio parahaemolitycus* derived from clinical samples has proposed links between acquisition of virulence and multiple drug resistance traits and intercellular transmissibility of mobile genetic elements in the environment. To date, very few information is available on environmental *Vibrio* isolates. In this study, we characterized eleven *Vibrio* strains bearing the SXT/R391-like integrative and conjugative elements (ICEs) derived from aquatic products and environment in the Yangtze River Estuary, China.

**Results:**

The eleven *Vibrio* strains were isolated in 2010 to 2011, and taxonomically identified, which included six *Vibrio cholerae*, three *Vibrio parahaemolyticus*, one *Vibrio alginolyticus* and one *Vibrio natriegens*. Most of the strains displayed strong resistance phenotypes to ampicillin, mercury and chromium. The majority of their ICEs, which belong to S and R exclusion system groups, contain ICEs-chromosome junction sequences and highly conserved core-genes required for ICE transfer. However, comparative sequence analysis uncovered interesting diversity in their mosaic accessory gene structures, which carry many novel genes that have not been described in any known ICEs to date. In addition, antibiotic resistance was transmitted by ICE*Vch*Chn6 and ICE*Vpa*Chn1 from *V. cholerae*, *V. parahaemolyticus* to *E. coli* MG1655 via conjugation, respectively. Our data also revealed that the ICEs characterized in this study are phylogenetically distant from most of the SXT/R391 ICEs reported previously, which may represent a novel cluster likely shaped by the ecological environment in the Yangtze River Estuary, China.

**Conclusions:**

This study constitutes the first investigation of ICEs-positive *Vibrio* spp. in the Yangze River Estuary, China. The newly identified ICEs were characterized with mosaic accessory gene structures and many novel genes. The results demonstrated self-transmissibility of antibiotic resistance mediated by two of the ICEs from *V. cholerae*, *V. parahaemolyticus* to *E. coli* via conjugation, respectively. Our results also revealed that the ICEs examined in this study may represent a novel cluster in the SXT/R391 family.

## Background

Integrative and conjugative elements (ICEs) are self-transmissible mobile genetic elements that mediate horizontal gene transfer between bacteria [1]. ICEs share certain features of phages, transposons and plasmids. But unlike these elements, ICEs integrate into and replicate as part of their host chromosomes, and can be transferred via conjugation [1,2]. ICEs and related elements can constitute a large proportion of bacterial chromosomes [3], and bestow a wide range of phenotypes upon their host with carried gene cassettes [4]. The first described ICEs-related elements were Tn916 from *Enterococcus faecalis* in 1980 [5] and CTnDOT from *Bacteroides thetaiotaomicron* in 1988 [6]. To date, a variety of ICEs have been classified into several families, and have been reported in diverse Gram-positive and Gram-negative bacteria [1,7], among which the SXT/R391 family were identified in *Vibrionaceae* isolates of clinical and environmental origins [8-10].

*Vibrionaceae* are Gram-negative, mesophilic and chemoorganotrophic bacteria, which belong to γ-proteobacteria. They are virtually ubiquitous in aquatic environments, including estuaries, marine coastal waters and sediments, and aquaculture settings worldwide [11]. Globally water-borne infectious diseases are one of the major contributors to disease burden and mortality [12]. Pathogenic *Vibrio cholerae* and *Vibrio parahaemolyticus* are serious human food-borne pathogens, causing cholera epidemics and diarrheal disease, respectively, and continue to be prevalent particularly in developing countries with disputable sanitary conditions [13]. The SXT element was originally discovered in *V. cholerae* O139, the first non-O1serogroup of *V. cholerae*, which gave rise to epidemic cholera in India and Bangladesh in early 1990s [14]. Unlike E1 Tor O1 strains of *V. cholerae,* the O139 stain was identified to harbor characteristic pattern of resistance to sulfamethoxazole, trimethoprim, streptomycin and furazolidone, which was carried on a ~100 kb self-transmissible SXT element [14]. Comparative sequence analysis revealed closer genetic relationship between the SXT and R391 element (89 kb) that was identified in *Providencia rettgeri* isolate in South Africa in 1972 [15,16]. Numerous previous studies revealed that all SXT/R391-related ICEs shared highly conserved module structures that encode mating-machinery for conjugation, and intricate regulatory systems to control their excision from the chromosome as well as their self-transmissibility [4]. The intercellular transmissibility of the mobile genetic elements with carried gene cassettes could constitute important driving forces in genome evolution and speciation of *Vibrios*, but also mediate the emergence, resurgence and spread of multiple drug resistant pathogens [17-19].

China has become the world’s largest producer of aquatic products since 2002 (People’s Republic of China, Fishery Products Annual Report). The East China Sea has been one of the major fishing grounds, especially within the Yangtze River plume and its surrounding sea along China’s coast [20]. Along with improved aquaculture production, however, incidences of food-borne illnesses caused by consumption of aquatic products contaminated with *Vibrios* have also rapidly increased, particularly in the littoral provinces [21]. Previous research suggested that acquisition of virulence and resistance traits through horizontal gene transfer might occur at high frequency through microbial contacts in the environment [22]. Nevertheless, to date, numerous studies have been conducted to identify ICEs-harboring *Vibrios* from clinical samples in different parts of the world [23], but very few information is available on environmental isolates. Thus, in this study, we focused on analyzing the *Vibrio* strains bearing the SXT/R391-related ICEs that were isolated from aquatic products and environment in the Yangtze River Estuary in Shanghai, China. Molecular structures of the ICEs and phenotypes of their hosts have been characterized. The information will facilitate the better understanding of possible mechanism underlying ICE evolution and dissemination of food-borne diseases mediated by the mobile genetic elements.

## Results and discussion

### Bacterial isolation, screening and identification of ICEs-positive strains

The Yangze River, being the third largest river (about 6,300 km in length) in the world, originates from the Qingzhang plateau, runs through eleven Chinese provinces and regions, and finally enters into the East China Sea in Shanghai, China. Environmental surface water samples were collected from the Yangtze River Estuary in Shanghai during the years between 2010 and 2011, while aquatic products including shrimps and fish were sampled from fish markets distributed in Shanghai in 2011. Pure cultures of *Vibrio* isolates were transferred into sterile 96-well microtiter plates, and used for PCR-based screening of the conserved essential integrase gene (*int*) of SXT/R391-related ICEs (see the Methods). A total of one hundred and fifty three isolates were detected positive for the *int* gene from about forty one plates. Among these, eleven isolates were assessed and reported in this study, for the ICEs-chromosomal junction sequences and or highly conserved core-genes required for ICE transfer tested in this study were identified.

Strain taxonomy assays yielded six *V*. *cholerae*, three *V. parahaemolyticus*, one *Vibrio alginolyticus* and one *Vibrio natriegens* strains (Table 1). All the *V*. *cholerae* strains were identified as non-O1/O139 serotypes, while the *V. parahaemolyticus* strains were identified as O5:KUT serotype. Toxin-related genes were detected by PCR. In all cases, *V. cholerae* strains were detected as not virulent, since amplification of cholera CT toxin *ctxA* gene was negative. Among the *V. parahaemolyticus* strains, all were detected positive for the *tlh* gene, but featured no toxic *tdh* and *trh* genes. The *V. alginolyticus* Chn4 was detected negative for the toxic *trh* gene, whereas *V. natriegens* yielded no products for the toxic genes tested.

**Table 1:** **Phenotypic resistance profiles for antibiotics and heavy metals of the *Vibiro *** **stains harboring SXT7R391-like ICEs isolated from aquatic products and environment in the Yangtze River Estuary**

**Strains**	**ICEs**	**Source, year of isolation**	**Resistance to antibiotics**	**Resistance to heavy metals**
*V. cholerae* Chn5^*^	ICE*Vch*Chn1	Yangze River Estuary, surface water, 2010-2011	STR	**-**
*V. cholerae* Chn64	ICE*Vch*Chn2	Yangze River Estuary, surface water, 2010-2011	AMP	Hg, Cd, Cu
*V. cholerae* Chn86	ICE*Vch*Chn3	Yangze River Estuary, surface water, 2010-2011	-	Hg, Cd, Cu
*V. cholerae* Chn91	ICE*Vch*Chn4	Yangze River Estuary, surface water, 2010-2011	AMP, RIF	Hg, Cd, Pb, Cu
*V. cholerae* Chn92	ICE*Vch*Chn5	Yangze River Estuary, surface water, 2010-2011	AMP, RIF	Hg, Cd, Zn, Pb, Cu
*V. cholerae* Chn108 ^*^	ICE*Vch*Chn6	Yangze River Estuary, surface water, 2010-2011	AMP, SUL, STR	Hg, Cd, Pb
*V. parahaemolyticus* Chn25^*^	ICE*Vpa*Chn1	Shanghai fish markets, shrimps, 2011	SUL, STR	-
*V. parahaemolyticus* Chn46	ICE*Vpa*Chn2	Shanghai fish markets, shrimps, 2011	AMP	-
*V. parahaemolyticus* Chn66	ICE*Vpa*Chn3	Shanghai fish markets, shrimps, 2011	AMP	Hg, Cd, Pb
*V. alginolyticus* Chn4	ICE*Val*Chn1	Shanghai fish markets, shrimps, 2011	AMP	Hg, Cd, Pb
*V. natriegens* Chn64	ICE*Vna*Chn1	Shanghai fish markets, shrimps, 2011	AMP, SUL, STR	-

*AMP* ampicillin, *RIF* rifampicin, *SUL* sulfamethoxazole, *STR* streptomycin, *Cd* chromium, *Cu* copper, *Hg* mercury, *Pb* lead, *Zn* zinc; -: not detected.

^*^The strains were employed as the donors in conjugation experiments.

### Antimicrobial susceptibility and heavy metal resistance of the *Vibrio* strains harboring the SXT/R391-like ICEs

The eleven *Vibrio* strains harboring the SXT/R391-like ICEs derived from aquatic products and environment in the Yangtze River Estuary were characterized by antimicrobial susceptibility testing. As summarized in Table 1, all strains were susceptible to five of the ten antimicrobial agents tested, including chloramphenicol, kanamycin, gentamicin, spectinomycin and trimethoprim. Strain *V. cholerae* Chn86 was susceptible to all the ten agents. It is known that ICEs transfer very diverse functions to allow their host to grow in hostile environments [4]. The SXT/R391-related ICEs lacking genes coding for antibiotic resistance were also found in some other *Vibrios*, such as others carrying ICE*Vch*Vie0 [8] and ICE*Vsc*Spa2 [10]. In addition, three strains exhibited resistance to sulfamethoxazole and streptomycin (Table 1), the typical resistance carried on SXT [14] and many other SXT/R391 elements [4,9,10]. Ampicillin resistance was the most predominant amongst the *Vibrio* strains examined in this study, most of which exhibited strong resistance phenotype (MIC ≥256 μg/ml) against this agent. This result correlates with that of Taviani et al. [9], where the majority of ICEs-positive *Vibrios* isolated from environmental water samples in Mozambique exhibited ampicillin resistance phenotypes [9]. It was supposed that the widespread of ampicillin-resistant bacteria may be attributed to the abuse of drugs and the inappropriate release of industrial wastes into environment [9]. However, compared with the *Vibrios* isolated from marine aquaculture environment in Spain and Portugal, which displayed multiple drug resistance to seven agents tested [10], our data revealed notable narrow resistance patterns yielded by the *Vibrios* of the Yangtze River Estuary origin.

Susceptibility of the strains to heavy metals including mercury (Hg), chromium (Cr), lead (Pb), zinc (Zn), and copper (Cu) was also determined (Table 1). About 70% of the strains displayed strong resistance to Hg (≥1 mM) and Cr (≥10 mM), half of which also showed high level of resistance to Pb (≥10 mM). Estuaries are zones of complex interaction between fluvial and marine process that act as geochemical trap for heavy metals [24,25]. Being located in one of the highest density of population and fastest economic developing areas in China, the Yangtze River Estuary area has suffered heavy metal contamination [26,27]. Our data in this study provide the first example of the high proportion of heavy metal resistant *Vibrios* in the Yangze River Estuary. Similarly, Hg resistance traits were also found in R391, ICE*Spu*PO1 [28], ICE*Vsp*Spa1 [10] and ICE*Eni*Spa1 [10], the latter two of which were isolated from marine aquaculture environments. In addition, four strains including *V. cholerae* Chn5, *V. parahaemolyticus* Chn25 and *V. natriegens* Chn64 were susceptible to all the heavy metals tested, while *V. cholerae* Chn92 was the only one showing low level of resistance to Zn. Although based on a fairly small number of isolates analyzed here, lower resistance percentage and level were detected from the strains isolated from aquatic products. The genes responsible for the resistance phenotypes of the *Vibrio* strains were further analyzed by sequence analysis of variable regions in the SXT/R391-like ICEs and conjugation experiments (see below).

### Features of the ICEs-chromosomal *attL* and *attR* junction sequences

**Figure 1:**
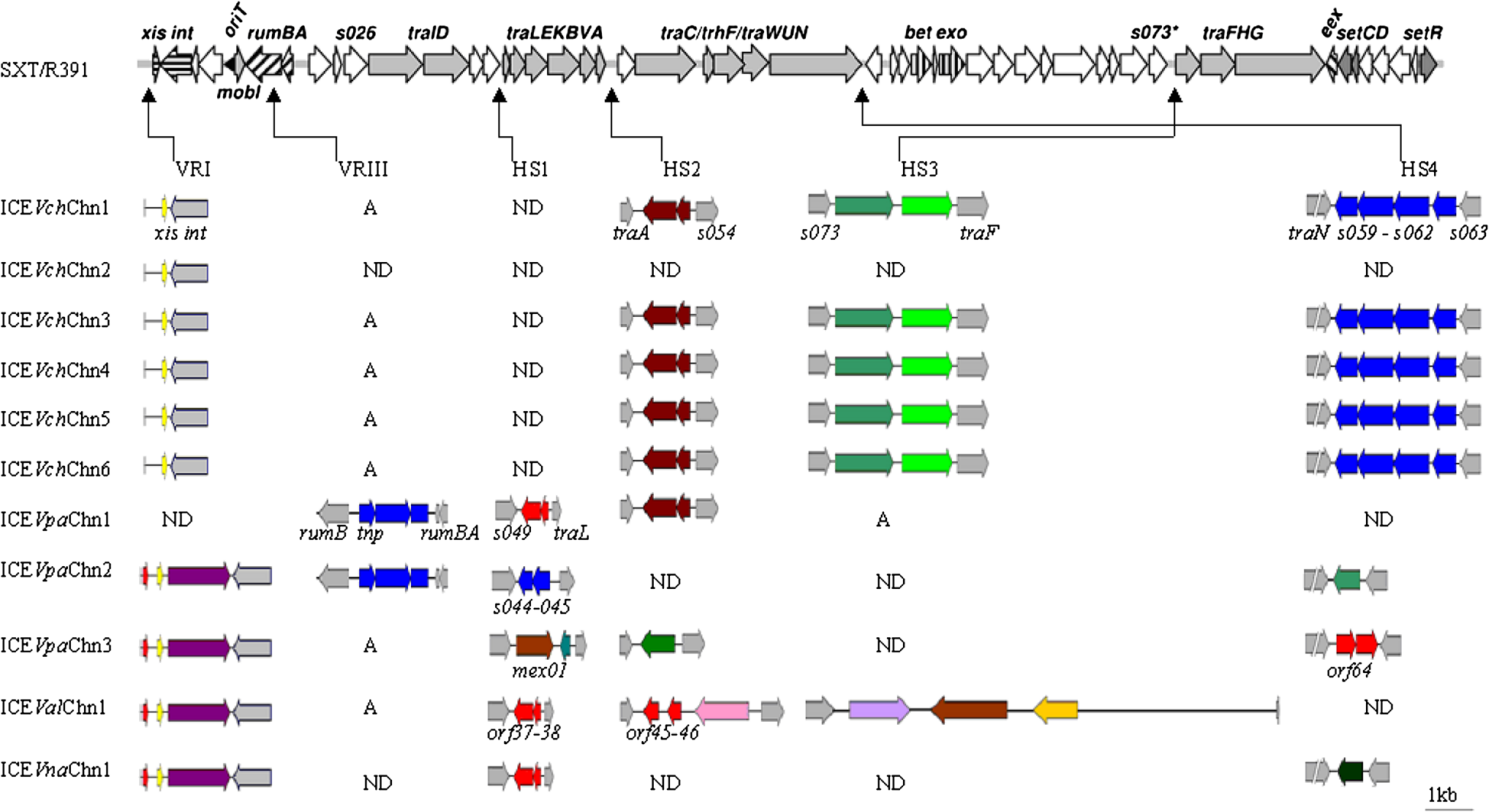
**Comparison of the accessory gene organizations in the ICEs characterized in this study with the other known SXT/R391 ICEs.** The gene organization of SXT/R391 ICEs was depicted by Wozniak et al. [23]. The genes that were inferred to encode homologous proteins were shown in the same colors in each variable and hotspot region. A, absence; ND, not detected.

Integrases of the SXT/R391 ICEs are members of the tyrosine recombinase family and mediate site-specific recombination between *attP* and *attB* sites on the circular form of ICEs and their respective host chromosomes, which yields the *attL* and *attR* ICE-chromosome junction sequences [4]. The sequences directly adjacent to the *attL* site (also known as variable region I, VRI) were amplified and determined from the ICEs characterized in this study. As illustrated in Figure 1, these sequences could form two distinct groups, except ICE*Vpa*Chn1. One of these with a 4.1-kb amplified fragment includes ICE*Vpa*Chn2, ICE*Vpa*Chn3, ICE*Val*Chn1 and ICE*Vna*Chn1 (GeneBank: KF411050). Unlike SXT and R391, these four elements have the same gene organization as the VRI sequence of ICE*Vch*Ind5, an ICE first detected in *V. cholerae* O1 in Sevagram, India, in 1994 (GenBank: GQ463142) [23]. They all consist of four previously described genes, encoding a conserved hypothetical protein, a recombination directionality factor (Xis), a DNA mismatch repair protein and an Int, respectively. The function of the hypothetical protein in ICE integration at *attL* site still remains unknown. The second group that yielded a 2.1-kb PCR product comprises six ICEs, and displays a SXT-specific molecular profile in the VRI [29], only containing the *xis* and *int* genes (GeneBank: KF411049). Existence of additional genes preceding the *int* genes in the vicinity of *attL* sites may suggest specific-integration mediated by Ints in these isolates [30].

To further characterize the ICEs, we also examined their right junction sites that generally locate in host chromosomal *prfC* genes, encoding a non-essential peptide release factor 3 in *E. coli, V. cholerae* and other hosts [31]. Amplification of *attR* sites achieved two outcomes. A predicted amplicon (0.3-kb) was detected from nine strains, characterizing recombination of circular ICEs into their respective host chromosomes. In addition, PCR amplification yielded no evidence for the presence of *attR* sites in ICE*Vpa*Chn3 and ICE*Vpa*Chn1. The latter also appeared to lack *attL* site. The integrity of *prfC* genes in their respective hosts was subsequently analyzed. Interestingly, *V. parahaemolyticus* Chn66 carrying ICE*Vpa*Chn3 was detected negative for an intact *prfC* gene, suggesting a possible ICE integration into this gene locus that resulted in a consequential variant *attR* junction sequence. An intact *prfC* gene was identified in *V. parahaemolyticus* Chn25 carrying ICE*Vpa*Chn1. Given that neither *attL* nor *attR* site seemed present in this strain, this result, coupled with the previous observation [9], argued for an additional integration site rather than the *prfC* gene in *V. parahaemolyticus* strains.

### Conserved-core genes in ICE modules

Although individual ICE has been found to bestow various phenotypes upon their host, all ICEs contain three modules that mediate their integration and excision, conjugation, and regulation [1]. To better characterize the ICEs identified in this study, besides the *int* and *xis* genes functioning in the maintenance module, we also examined *traI, traC, traG* and *setR* genes that belong to a highly conserved minimal gene set required for ICE transfer [1,9]. In the dissemination module, the *traI* gene encodes a relaxase and participates in ICE DNA processing and single-stranded DNA mobilization to the recipient cell [32]. Amplification of the *traI* gene yielded a desired 0.7-kb amplicon from all the ICEs except ICE*Vch*Chn2. Similarly, the *traC* and *traG* genes encoding typical conjugation transfer proteins involved in mating-pair formation were also examined by PCR. In all cases, both *traC* and *traG* genes were detected positive. Sequences of the *traI, traC* and *traG* amplicons were determined, and BLAST analysis showed 89-94%, 95-100% and 93-99% sequence identity at the amino acid level to the corresponding proteins of SXT, respectively. In the regulation module, the *setR* gene inhibits the expression of *setDC* operon that encodes the master transcriptional activators required for SXT transfer [33]. As an important regulator, the *setR* gene was thus examined. Except ICE*Vna*Chn1, a predicted 0.9-kb amplicons was yielded from all the ICEs tested, which shared 99-100% amino acid sequence identity to the SetR of SXT.

### Evolution origin of the SXT/R391-like ICEs

**Figure 2:**
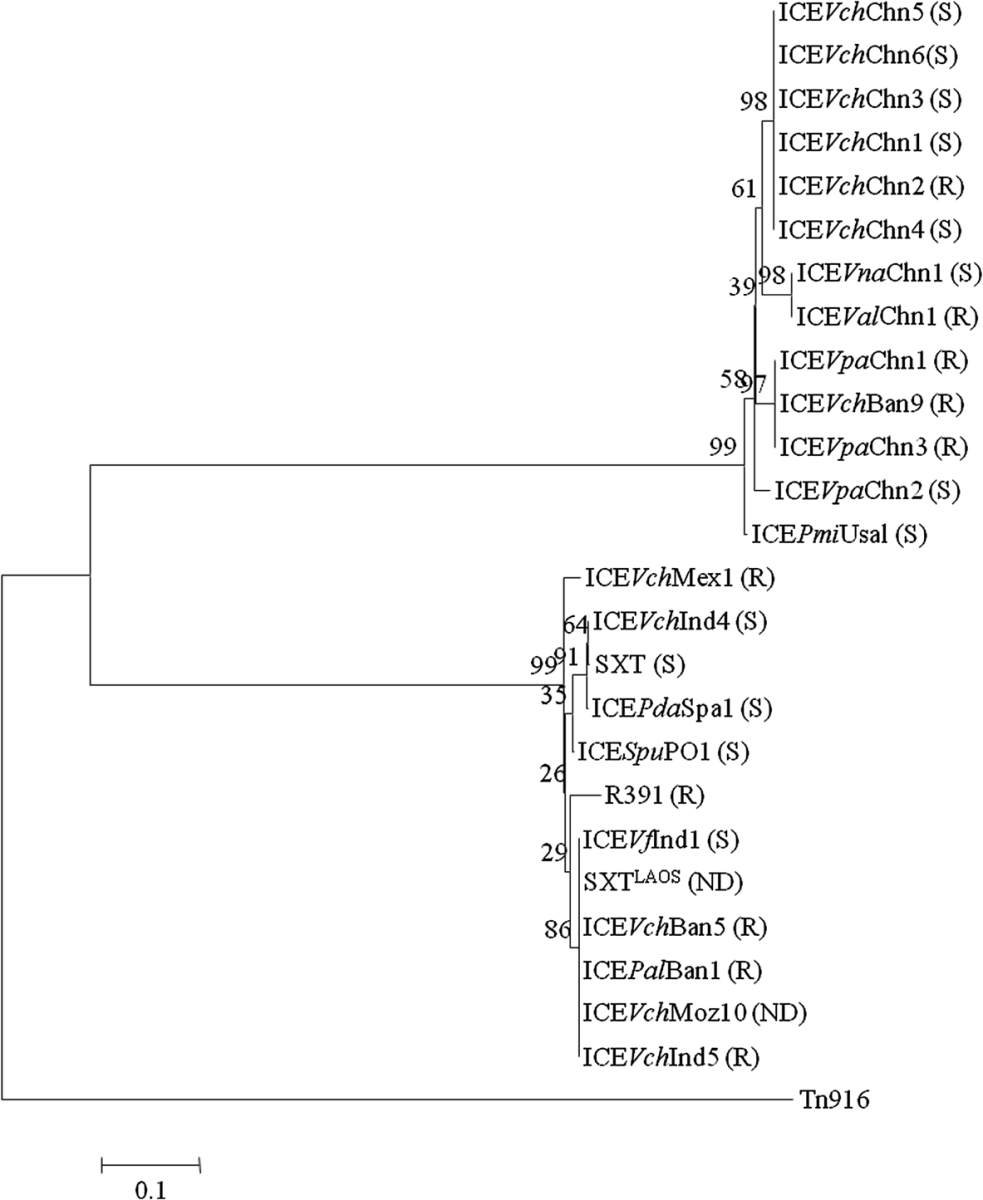
**Phylogenetic tree showing evolutionary relationship of the ICEs harbored by the *Vibrio *spp. isolated from aquatic products and environment in the Yangze River Estuary, China.** Based on the *int* gene sequences derived from the ICEs characterized in this study and from some known SXT/R391 and Tn916 ICEs in the public databases, the neighbor-joining phylogenetic tree was constructed by using the MEGA 4.0. The accession numbers in the GenBank database of the ICEs displayed in the phylogenetic tree are as following: SXT: AY055428; R391: AY090559; ICE*Vch*ind4: GQ463141; ICE*Pda*Spa1: AJ870986; ICE*Vfl*Ind1: GQ463144; SXT^LAOS^: AB114188; ICE*Vch*Mex1: GQ463143; ICE*Spu*PO1: CP000503; ICE*Vch*Ban9: CP001485; ICE*Pal*Ban1: GQ463139; ICE*Vch*Ban5: GQ463140; ICE*Vch*Ind5: GQ463142; ICE*Pmi*Usa1: AM942759; ICE*Vch*Moz10: NZ_ACHZ01000012.1 and Tn916: EF432727.1. Bootstrap percentages are shown at nodes. The scale bar represents 0.1 changes per amino acid. R and S represent R and S exclusion groups, respectively. ND: not detected.

Based on the *int* gene sequences derived from the ICEs analyzed in this study and a selected set of its homologs from SXT/R391 ICEs identified in the public databases, a phylogenetic tree was constructed by the MEGA4.0. It revealed that these ICEs could form two distinct clusters, designated I, and II (Figure 2). Remarkably, the majority of the previously reported ICEs derived from clinical and environmental *Vibrios* and other species were distributed in Cluster I, whereas all the ICEs obtained in this study fell into Cluster II. Interestingly, phylogenetic analysis showed closely related relationship between the ICEs of the Yangze River Estuary origin and two of previously reported ICEs, ICE*Vch*Ban9 and ICE*PmI*Usa1. The former was isolated from clinical *V. cholerae* O1 strain in Bangladesh [34], while ICE*PmI*Usa1 was identified in clinical *Proteus mirabilis* strain isolated from USA [35]. Despite different environmental origins, this result may suggest a common ancestor shared by these ICEs in their evolutionary histories.

### Hotspots in the SXT/R391-like ICEs

Accessory genes that are not required for transmission or other core ICE functions are restricted to insert into particular loci in several ICE families [1]. The SXT/R391-related ICEs contain five hotspots for insertion, where the boundaries between conserved and variable DNA are generally conserved [23]. DNA insertions in four hotspots (HS1 to HS4) that are related with resistance determinants and other characterization in previous reports were analyzed in the ICEs identified in this study.

Hotspot1. Amplification and sequencing of hotspot1 yielded the evidence for different DNA insertions into HS1 loci in the ICEs analyzed here. Their gene organization is presented in Figure 1. About 0.7-kb DNA insertion was identified in ICE*Vpa*Chn1, ICE*Val*Chn1 and ICE*Vna*Chn1, respectively. They all encode two conserved hypothetical proteins with unassigned gene functions in the public databases (GenBank: KF411051-411053), which display high sequence identities (94-98%) at the amino acid level to the orf38 and orf37 in the HS1 of R391 (GenBank: AY090559). Similarly, ICE*Vpa*Chn2 carries a 0.8-kb inserted sequence in the HS1 (GenBank: KF411054). Sequence analysis showed identical gene content to the SXT HS1, which consists of the previously described *s044* and *s045* genes encoding putative toxin-antitoxin system proteins [23]. Interestingly, a mosaic sequence structure was identified from the HS1 (GenBank: KF411055) of ICE*Vpa*Chn3. Half of the DNA insertion (2.0-kb) contains a homologous gene to *mex01* that occurs in the HS1 of ICE*Vch*Mex1 [36], encoding a putative Fic (filamentation induced by cAMP) family protein (GenBank: ACV96444.1) involved in cell division. On another half, a novel gene was identified that has not been described in any ICEs to date. Its closest match (94% amino acid identity) was a plasmid maintenance system antidote protein (NCBI Reference Sequence: ZP_11329092.1) of the *Glaciecola polaris* LMG 21857. Additionally, in the remaining six ICEs, PCR amplification with the HS1-F/R primers (Table 2) was negative, implying the variance of boundary genes that may result from gene recombination, or the presence of large DNA insertions that may not be amplified by the PCR conditions used in this study.

**Table 2:** Oligonucleotide primers used in this study

**Primers**	**Sequence 5′-3′**	**Target genes or regions**	**References**
27F	AGAGTTTGATCCTGGCTCAG	16S rDNA	46
1492R	GGTTACCTTGTTACGACTT		
attL-F	ATGACCAACCAGAACttyytnmayga	*attL-int*	This study*
attL-R	ttywsnttyraCCCGGACCCGG		
Int-F	GACGCATTTCATCCAGG	*int*	This study
Int-R	GCAACAGCGGGTAGACA		
attR-F	GGTTTAGCCACAGTTGTTC	*attR*	This study
attR-R	CGTCAGGGTGCGAGAT		
PVP-F	GGCAATGGAAAAGAAGACGCGA	*prfC* of *V. parahaemolyticus*	9
PVP-B	ATCAAACTCAGGACATGCACCC		
PVC-F	TCCTGCACCTTGCTCTGCTCT	*prfC* of *V. cholerae*	9
PVC-B	ACCACGCTCTTTTTCCATTTCCAT		
setRpF	CGGCGGAGATGTTTTTGT	*setR*	8
setRpR	GTGCGCCAATGCTCAGTT		
traC-F	TGACGCTGTTTTCACCAACG	*traC*	8
traC-B	GGCACGACCTTTTTTCTCCC		
traI-F	GCAAGTCCTGATCCGCTATC	*traI*	8
traI-R	CAGGGCATCTCATATGCGT		
LEFTF3	GGTGCCATCTCCTCCAAAGTGC	*rumBA* (VRIII)	39
RUMA	CGAGCAATCCCCACATCAAG		
HS1-F	GGTTCAGGCGTCATCTT	*s043-traL*	This study
HS1-R	TCTCATCGGCACTCCA		
HS2-F	GTCGTTGCCAGCACTCA	*traA-s054*	This study
HS2-R	CGCCAGAATGATTGGAGAT		
HS3-F	GGTGTACTGGAAGACCGG	*s073-traF*	This study
HS3-R	CAGGCAGCACTGAAAGG		
HS4-F	AGTGACCCAGGCATAGAC	*traN-s063*	This study
HS4-R	GAAGAGGAAACAGATAACCC		
E1	TTGCGGGAGATTATGCTC	*eex*	43
E2	TGACCATCAATGAAGGTTG		
T1	CATCTAGCGCCGTTGTTAATCAGGT	*traG*	43
T2	ATCGCGATACTCAGCACGTCGTGAA		
ctxA-F	CGGGCAGATTCTAGACCTCCTG	*ctxA*	48
ctxA-R	CGATGATCTTGGAGCATTCCCAC		
L-TLH	AAAGCGGATTATGCAGAAGCACTG	*tlh*	47
R-TLH GCT	ACTTTCTAGCATTTTCTCTGC		
tdh-1	CCATCTGTCCCTTTTCCTGCC	*tdh*	47
tdh-4c	CCACTACCACTCTCATATGC		
VPTRH-L	TTGGCTTCGATATTTTCAGTATCT	*trh*	47
VPTRH-R	CATAACAAACATATGCCCATTTCCG		
P1	TGCTGTCATCTGCATTCTCCTG	circular ICEs	24
P2	GCCAATTACGATTAACACGACGG		

*The primers were designed based on the corresponding gene sequences of SXT (GenBank: AY055428).

Hotspot2. In addition to SXT or R391-specific molecular profiles in hotspot2 loci as previously reported [23], variable gene contents in HS2 were identified in eight ICEs characterized in this study (Figure 1). Previous studies indicated that most SXT/R391 ICEs contain *mosA* and *mosT* genes in HS2, which encode a novel toxin-antitoxin pair that promotes SXT maintenance by killing or severely inhibiting the growth of cells that have lost this element [37]. However, the two genes were absent from the HS2 (1.3 kb) in six ICEs including ICE*Vch*Chn1, ICE*Vch*Chn3, ICE*Vch*Chn4, ICE*Vch*Chn5, ICE*Vch*Chn6 and ICE*Vpa*Chn1. These results are consistent with those yielded from R391 and few other ICEs [10,37]. Nevertheless, BLAST analysis of the HS2 (GenBank: KF411056-KF411060) in these six elements revealed that they contain two homologous genes (98% amino acid identity) to those that occur in the 3′-region of the HS2 in ICE*Vsp*Por2, possibly encoding additional anti-toxin component protecting against the loss of the ICEs [10]. It is thus interesting to study if these two genes could compensate for the *mosAT* loss in these elements. In this study, BLAST analysis also revealed that ICE*Val*Chn1 (GenBank: KF411061) contains the first two (*orf45, orf46*) of ten genes in the HS2 of R391. However, unlike R391, downstream of these two genes, ICE*Val*Chn1 also contains a gene with 98% amino acid sequence identity to a transposase of IS605 OrfB family of the *Shewanella sp*. ANA-3 (GenBank: ABK50401), which could indicate gene recombination in the HS2 of ICE*Val*Chn1 possibly mediated by this transposase. In addition, ICE*Vpa*Chn3 shows a 5′-region truncated version of the HS2 of ICE*Vch*Mex1 [36], and contains a homologous gene to previously described *mex02* (98% amino acid identity) (GenBank: KF411062). Finally, amplification of the HS2 yielded no PCR product from ICE*Vch*Chn2, ICE*Vpa*Chn2 and ICE*Vna*Chn1, which may resulted from large DNA insertions, e.g. a 29.2-kb insertion in the ICE*Spu*PO1 HS2 carrying heavy metal efflux gene clusters [28].

Hotspot3. Transposon-like structures carrying genes involved in trimethoprim resistance or DNA modification, recombination or repair in diverse putative restriction-modification systems were found within the hotspot3 [23]. As illustrated in Figure 1, about 5.4-kb DNA insertion was identified in five ICEs including ICE*Vch*Chn1, ICE*Vch*Chn3, ICE*Vch*Chn4, ICE*Vch*Chn5 and ICE*Vch*Chn6, respectively. BLAST analysis revealed the same gene content as that in the HS3 of SXT^LAOS^[38], encoding an exonuclease and a helicase (99% amino acid identity) (GenBank: KF411063). In addition, a large DNA fragment was amplified from the HS3 (GenBank: KF411064) of ICE*Val*Chn1. It is 9.7 kb in length and shows no significant similarity in gene content with any known ICEs that have been characterized to date. Database searches revealed that besides the boundary genes, the DNA insertion contains at least three more genes, encoding a putative glucose-1-phosphate adenylyltransferase and a RNA-directed DNA polymerase, displaying high sequence identities (60-100%) at the amino acid level to corresponding homologs in the genomes of *Vibrios* and closely related species in the public databases. It also contains a novel gene with 76% amino acid sequence identity to a transposase of the *Vibrio metschnikovi* CIP 69.14 (GenBank: eex38460.1). Moreover, BLAST search yielded no significant similarity in its 3′-region sequence of the insertion, almost half of its full length, indicating completely novel genes carried by this ICE. Finally, ICE*Vpa*Chn1 harbored no DNA insertion in the HS3, from which only the boundary gene sequences were amplified, while four ICEs including ICE*Vch*Chn2, ICE*Vpa*Chn2, ICE*Vpa*Chn3 and ICE*Vna*Chn1 failed to yield any PCR products in their respective HS3 locus.

Hotspot4. Extensive differences in molecular profiles of hotspot4 were reported in the SXT/R391 ICEs [23]. Amplification and sequencing of the HS4 yielded about 5.6-kb inserted sequence from five ICEs (Figure 1). Database searches showed the SXT-specific molecular profile in their respective HS4 site (GenBank: KF411068). These elements contain three homologous genes (94-100% amino acid identity) to previously described *s060* to *s062* in the SXT HS4, encoding a putative nuclease and two conserved hypothetical proteins of unknown functions in the current literature. Similarly, ICE*Vpa*Chn3 has R391-specific genes *orf64* in the HS4 (2.9 kb), encoding a conserved hypothetical protein (GenBank: KF411065). In addition, ICE*Vpa*Chn2 displays a truncated molecular profile of the ICE*Pda*Spa1 HS4, containing the *spa06* gene coding for a conserved hypothetical protein (GenBank: KF411066), while ICE*Vna*Chn1 harbors a novel gene in a 3.6-kb inserted sequence in the HS4 (GenBank: KF411067). Its closest match (99-67% amino acid identity) was a conserved hypothetical protein with unknown function in different bacteria including *Pasteurella*, *Shewanella* and *Salmonella*. Finally, no PCR product was yielded from the HS4 of ICE*Vch*Chn2, ICE*Vpa*Chn1 and ICE*Val*Chn1, respectively.

Although DNA insertions were identified in four hotspots of the ICEs characterized in this study, remarkably, many genes carried by these sequences are predicted to encode conserved hypothetical proteins whose functions had not been assessed in the public databases. Nevertheless, based on sequence analysis, some DNA insertions are assumed to confer an adaptive function upon their hosts with carried gene cassettes. For example, the DNA sequences inserted into HS3 loci of ICE*Vch*Chn1, ICE*Vch*Chn3, ICE*Vch*Chn4, ICE*Vch*Chn5 and ICE*Vch*Chn6 carry genes encoding putative helicases and exonucleases. Such genes may provide the host with barriers to invasion by foreign DNA and/or promote the integrity of ICEs during its transfer between hosts [23].

### Variable region III in the SXT/R391-like ICEs

Antibiotic resistance determinants are clustered into the *rumB* gene (known as variable region III, VRIII) in many SXT/R391 ICEs, such as R391, SXT, ICE*Vch*Ban5 and ICE*Vch*Ind5 [23,39,40]. Amplification of the VRIII yielded two groups of PCR products from the ICEs analyzed in this study. A predicted 0.8 kb-product was detected in seven ICEs, indicating the absence of any gene insertion into the *rumB* gene. Similar results were also reported in several other ICEs, such as ICE*Vsc*Spa1-3, ICE*Eni*Spa2 and ICE*Sha*Por1 [10], all of which contains an intact *rumB* gene in their respective VRIII. Additionally, a 3.9-kb inserted sequence (GenBank: KF411069) was identified in ICE*Vpa*Chn1 and ICE*Vpa*Chn2, respectively (Figure 1). BLAST searches revealed that these two elements contain three homologous genes (97-99% amino acid identity) to the previously described *tnp*, *tnpA* and *s021* that occur in the VRIII of ICE*Vsp*Spa2 [10] and ICE*Vch*Vie0 [8], showing a truncated copy of the VRIII in SXT [16]. The three genes are predicted to encode two putative transposases and a methyl-directed mismatch DNA repair protein. This result perhaps suggests a common evolutionary driving force shared by these ICEs in the HS4 loci possibly mediated by the transposases in the VRIII. Finally, no PCR product was yielded from the VRIII of ICE*Vch*Chn2 and ICE*Vna*Chn1, suggesting possible presence of large DNA insertions, e.g. 17.2 kb in SXT, which may not be amplified by the PCR conditions used in this study.

Most ICEs analyzed in this study were characterized by the absence of the typical antibiotic resistance gene clusters inserted into the *rumB* gene. Moreover, none of these resistance genes was detected to lay within the HSs under our analysis conditions, such as the *dfrA1* cassette in HS3 in four previously reported ICEs [23,39]. However, we cannot rule out the possibility of resistance determinants present elsewhere in the ICEs or in host genomes independently of ICE sequences. The former hypothesis seems more likely, for the successful transmissibility of the antibiotic resistance (Sul^r^ and Stp^r^) between two *Vibrio* strains *V. cholerae* Chn108 and *V. parahaemolyticus* Chn25 and *E. coli* MG1655 has been demonstrated by conjugation experiments (see below). The *rumB* and *rumA* genes encode a UV repair DNA polymerase and a UV repair protein, respectively [41]. Environmental strains tend to conserve ICEs devoid of antibiotic resistance genes by keeping a functional *rumBA*, compared with clinical strains not exposed to UV but to antibiotics [9]. Moreover, most of the ICE antibiotic resistance genes are found within transposon-like structures [23]. These may serve as a good explanation as to why typical antibiotic resistance gene clusters were not detected in the VRIII of the ICEs characterized in this study.

### Exclusion system

Entry exclusion systems specifically inhibit redundant conjugative transfers between cells that carry identical or similar elements [42,43]. SXT and R391 carry genes for an entry exclusion system mediated by two inner membrane proteins, TraG and Eex, which are expressed in the donor and recipient cells, respectively [44]. Consistent with previous results [10,43], the ICEs characterized in this study fell into two exclusion groups, S and R (Figure 2). Multiple sequence alignments revealed that the S group elements encode EexS proteins with typical exclusion sequences [45] in their carboxyl termini as known EexS proteins in public databases (data not shown). They also encoded TraG_S_ proteins with exclusion determinant residues P-G-E [43]. In contrast, four elements including ICE*Vch*Chn2, ICE*Vpa*Chn1, ICE*Vpa*Chn3 and ICE*Val*Chn1 fell into the R group, which encode the EexR, and TraG_R_ proteins with characteristic exclusion T-G-D residues (data not shown). It was reported that R391 and pMERPH, belonging to the R exclusion group, contain a DNA insertion conferring resistance to mercury immediately downstream of their respective *eexR* and *eexR4* genes [29,45]. Unexpectedly, in our study, neither the R nor the S group strains that display strong mercury resistance phenotypes was detected to carry any inserted sequence between the *eeX* and *traG* genes under our analysis conditions. The results suggest that the mercury resistance determinants or heavy metal efflux pumps mediating the resistance phenotypes may be present in additional loci in the ICEs, or in their host genomes independently of the ICE sequences. The latter hypothesis seems more likely based on the conjugation experiments.

### Conjugative capability

Four *Vibrio* stains with appropriate antibiotic selective markers were further analyzed as donors in conjugation experiments, except *V. natriegens* Chn64 carrying ICE*Vna*Chn1 with a deficient *traI* gene. The assay was facilitated by the finding that the donor strains that harbor ICE*Vch*Chn6 or ICE*Vpa*Chn1 were sensitive to chloramphenicol (Chl^s^), but resistant to streptomycin (Stp^r^) and sulfamethoxazole (Sul^r^), while the donor stain carrying ICE*Vch*Chn1 shows the Chl^s^ Stp^r^ phenotype (Table 1). Thus, we could use these antimicrobial agents to select for transconjugants. Moreover, the circular extrachromosomal form of these ICEs was detected positive by PCR analysis. Other *Vibrio* spp. strains without appropriate antibiotic selective markers were not analyzed in the conjugation testing, e.g. ICE*Vpa*Chn3 showing ampicillin resistant. Because the recipient cells also displayed ampicillin and rifampicin resistant phenotypes, these drugs can not be used to select transconjugants. Mating assays yielded the evidence for the successful conjugative transfer of ICE*Vch*Chn6 from *V. cholerae* Chn108 into the recipient *E. coli* MG1655 (Chl^r^, Sul^s^, Stp^s^) cells. Either Sul^r^ and Chl^r^, or Stp^r^ and Chl^r^ transconjugants were both obtained at a transfer frequency of about 2.9 × 10^-6^. Transconjugants were confirmed to integrate into the *prfC* gene of the *E. coli* MG1655 by colony PCR-based assays. However, among the conserved core-genes detected in this study, the *traI* gene seems deficient in the transconjugants, perhaps suggesting an integrated form of this mobile element on the recipient chromosome under the selective pressure. In addition, mating assays also demonstrated active self-transmissible function of ICE*Vpa*Chn1 from *V. parahaemolyticus* Chn25 into *E. coli* MG1655. Transconjugants with Sul^r^ and Chl^r^, as well as Stp^r^ and Chl^r^ phenotypes were both obtained at a similar transfer frequency with that of ICE*Vch*Chn6. However, unlike ICE*Vch*Chn6, all the conserved core-genes tested in this study were detected positive for the transconjugant *E. coli* MG1655 carrying ICE*Vpa*Chn1. It is not clear at this point about the alternative integration site in this donor genome. However, it was an ICE element, not a plasmid that transferred the antibiotic resistance between the donor and the recipient strains, because the major conserved-core genes in ICE modules and variable regions of ICEs were detected in the transconjugants. Finally, no transconjugant was observed on selective agar plates when *V. cholerae* Chn5 carrying ICE*Vch*Chn1 was employed as a donor in mating assays. Similarly, conjugation experiments yielded no evidence for the heavy metal resistance transfer mediated by the ICEs, the possible mechanism of which was discussed previously.

## Conclusions

This study constitutes the first investigation of ICEs-positive *Vibrio* spp. derived from aquatic products and environment in the Yangze River Estuary, China. The strains were taxonomically identified, which included six *V. cholerae*, three *V. parahaemolyticus*, one *V. alginolyticus* and one *V. natriegens*. Most of the strains displayed narrow antibiotic resistance patterns, but strong resistance phenotypes to ampicillin, mercury and chromium. Comparison of the ICEs characterized in this study with other known elements provided further evidence for the presence of extensive genetic recombination amongst SXT/R391 ICEs, which lead to three major molecular snapshots. Firstly, none of the ICEs analyzed here displays identical gene organization patterns in all variable regions tested as those of the previously reported ICEs. The results reinforce the finding yielded from the phylogenetic analysis in that these ICEs may represent a novel cluster in the SXT/R391 family, which could be shaped by the ecological environment in the Yangtze River Estuary, China. Secondly, distinct mosaic accessory gene structures with diverse origins are present in the ICEs characterized in this study. For example, the ICEs derived from aquatic products share accessory genes with those of clinical, environmental and aquaculture environmental origins in different parts of the world. On the other hand, similar foreign DNA appears to be captured by the ICEs in different environments. Finally, even within one hotspot, mosaic gene structures are present in some ICEs, such as the hybridized HS1 sequence in ICE*Vpa*Chn3. In addition, our results also demonstrated self-transmissibility of antibiotic resistance mediated by ICE*Vch*Chn6 and ICE*Vpa*Chn1 from *V. cholerae*, *V. parahaemolyticus* to *E. coli* via conjugation, respectively.

## Methods

### Bacterial isolation, screening and identification of ICEs-positive strains

Bacterial isolation was carried out according to the instructions of the China Government Standard (GB17378-2007) and the Standard of the Bacteriological Analytical Manual (BAM) of U.S. Food and Drug Administration (8th Edition, Revision A, 1998). Pure cultures of *Vibrio* isolates grown on selective thiosulfate citrate bile sucrose (Beijing Luqiao technology Co. Ltd., China) agar plates were picked, and transferred into sterile 96-well microtiter plates according to the instruction of the BAM. Bacterial cells in each row (12 wells) were combined and harvested for genomic DNA extraction and PCR-based screening of the conserved essential integrase gene (*int*) of SXT/R391-related ICEs. The isolates in the *int* gene*-*positive samples were further individually screened by PCR using the lysis buffer for microorganism to direct PCR kit (TaKaRa Biotechnology Co. Ltd. Dalian, China).

Strain taxonomy was carried out by conventional biochemistry tests and 16S rRNA gene amplification and sequencing with the primer pair 27F and 1492R [46] (Table 2). Serotypes were identified using the *V. cholerae* and *V. parahaemolyticus* specific diagnostic antiserum kits (Tianjin Biochip Co. Ltd., Tianjin, China). Toxin-related genes were detected by PCR using the primers previously described [47,48] and listed in Table 2.

### PCR conditions

Genomic DNA was prepared using MiniBest bacterial genomic DNA extraction kit ver.2.0 (TaKaRa). The concentration of DNA in the samples was determined using a multi-mode microplate reader BioTek Synergy™ 2 (BioTek Instruments, Inc., VT, USA). PCR amplification was performed in a 20 μl reaction volume containing 1 × Premix Ex Taq version (TaKaRa), 5 μM each of the oligonucleotide primers, and 5–10 ng of template DNA. The PCR amplification of the *int* gene was carried out with the primers Int-F and Int-R (Table 2) under the following conditions: initial denaturation of 95°C for 300s was followed by 30 cycles consisting of denaturation at 94°C for 30 s, primer annealing at 55°C for 30s, and elongation at 72°C for 1 min, followed by final elongation at 72°C for 5 min. The other PCR reactions were performed with appropriate annealing temperatures and elongation time according to melting temperatures of primer pairs and predicted lengths of PCR products. Long-range PCR amplification was performed using Takara LA Taq kit (Takara) according to the manufacturer’s instruction. All amplifications were performed in a Mastercycler® pro PCR thermal cycler (Eppendorf, Hamburg, Germany). A sample (5 μl) of each amplification reaction was analyzed by agarose gel electrophoresis. Amplified DNA fragments were visualized under short-wavelength UV light (260 nm) and imaged by UVP EC3 Imaging systems (UVP LLC, CA, USA).

The *attL* and *attR* junction sequences and hotspots (HS1 to HS4) of the ICEs analyzed in this study were individually amplified by PCR with the designed primer pairs complementary to the corresponding sequences and boundary genes of SXT (GenBank: AY055428) (Table 2). The *prfC*, *traI*, *traC*, *setR*, *traG*, *eex*, *rumBA* genes and the circular extrachromosomal form of the ICEs were individually amplified with the primers described in the literature [8,9,31,39,43] (Table 2).

### Sequence analyses

Automated DNA sequencing was carried out using ABI 3730XL capillary sequencer (Applied Biosystems, CA, USA) and BigDye® terminator version 3.1 cycle sequencing kit (Perkin-Elmer, MA, USA) at the China Human Genome Centre (Shanghai, China). Oligonucleotide primers were synthesized by Shanghai Sangon Biological Engineering Technology and Services Co., Ltd. (Shanghai, China). The sequences from complementing DNA strands were determined, and assembled into full length contigs by using the ContigExpress software (http://www.contigexpress.com). Putative functions were inferred by using the Basic Local Alignment Search Tool (BLAST) (http://ncbi.nlm.nih.gov/BLAST) and ORF finder (http://www.ncbi.nlm.nih.gov/projects/gorf). Multiple sequence alignments were performed using the ClustalW2 software (http://www.ebi.ac.uk/Tools/msa/clustalw2) [49]. The neighbor-joining method in the molecular evolutionary genetic analysis software package MEGA (version 4.0) [50] was used to construct a phylogenetic tree. A bootstrap analysis with 1000 replicates was carried out to check the reliability of the tree. The DNA sequences and annotation of the representative variable regions of ICE elements identified in this study have been deposited in GenBank database under the accession numbers from KF411049 to KF411069.

### Susceptibility to antimicrobial agents and heavy metals

The isolates were measured for *in vitro* susceptibility to antimicrobial agents according to the guidance of the Performance Standards for Antimicrobial Disk Susceptibility Tests of the Clinical and Laboratory Standards Institute (CLSI) (2006, Approved Standard-Ninth Edition, M2-A9, Vol. 26 No.1). Mueller-Hinton agar medium (Oxoid, UK), and the discs (Oxoid, UK) were used in this study. Examined antimicrobial agents included: 10 μg ampicillin (AMP), 30 μg chloramphenicol (CHL), 10 μg streptomycin (STR), 10 μg gentamicin (CN), 30 μg kanamycin (KAN), 5 μg rifampicin (RIF), 100 μg spectinomycin (SPT), 30 μg tetracycline (TET), 5 μg trimethoprim (TM), and 25 μg SXT (sulfamethoxazole (23.75 μg)-trimethoprim (1.25 μg). The assays were performed in triplicate experiments, and reference strain *Escherichia coli* ATCC25922 was purchased from the Institute of Industrial Microbiology (Shanghai, China) and used for quality control. Broth Dilution Testing (microdilution) was used to measure quantitatively the minimal inhibitory concentration (MIC) *in vitro* of the tested antimicrobial agents against the stains, according to the Methods for Dilution Antimicrobial Susceptibility Tests for Bacteria That Grow Aerobically (2006, CLSI, Approved Standard-Seventh Edition, M7-A7, Vol.26 No.2). Similarly, the MICs of the heavy metals, including Hg(NO_3_)_2,_ Cd(NO_3_)_2,_ Pb(NO_3_)_2_ and ZnCl_2_ (Sigma-Aldrich, USA), as well as CuSO_4_ (Songong, China), were also determined.

### Conjugation

Conjugation experiments were performed using the strains with appropriate selective markers as the donors (Table 1) and a chloramphenicol-resistant stain of *E. coli* (stain MG1655, a gift from Dr. Liping Zhao) as the recipient, according to the method described by Waldor et al. [14] with slight modification. The antimicrobial agents used for selection in plate mating assays included: chloramphenicol (30 μg/ml), sulfamethoxazole (128–160 μg/ml), streptomycin (30–60 μg/ml). Briefly, recipient and donor strains were individually cultured to log-phase, the latter was treated with mitomycin C (50 ng/ml) for 1 h at 37°C to increase transfer frequency of SXT elements (Beaber et al., [36]). Cell cultures were harvested by centrifugation, and mixed at a ratio of approximately 1:1. The cell mixture was resuspended in 0.2 ml LB, and then spotted onto LB agar plates. Mating was performed at 37°C for 6 h. Cells from the mating plates were harvested in 200 μl LB broth, and serial dilutions were spread onto the appropriate selective agar plates. The successful transfer of ICEs into the recipient strain was confirmed by colony PCR using the primers for characterizing the ICEs in this study (Table 2). The transfer frequency was calculated as the number of tansconjugants in mating cell mixture per donor cell. Similarly, heavy metal resistance was also used as selective marks in the conjugation experiments.

## Competing interests

The authors declare that they have no competing interests.

## Authors’ contributions

BL, YP and LC participated in the design of the study; YS and PY carried out the major experiments; YS, PY, BL, YP, XZ, CJ, YZ and LC analyzed data; LC drafted the manuscript, and HW revised it for important intellectual content and improvement. All authors read and approved the final manuscript.
